# First Detection of SARS-CoV-2 Delta (B.1.617.2) Variant of Concern in a Dog with Clinical Signs in Spain

**DOI:** 10.3390/v13122526

**Published:** 2021-12-16

**Authors:** Leira Fernández-Bastit, Jordi Rodon, Edwards Pradenas, Silvia Marfil, Benjamin Trinité, Mariona Parera, Núria Roca, Anna Pou, Guillermo Cantero, Cristina Lorca-Oró, Jorge Carrillo, Nuria Izquierdo-Useros, Bonaventura Clotet, Marc Noguera-Julián, Julià Blanco, Júlia Vergara-Alert, Joaquim Segalés

**Affiliations:** 1 Institut de Recerca i Tecnologia Agroalimentàries (IRTA), Centre de Recerca en Sanitat Animal (CReSA, IRTA-UAB), Campus de la UAB, 08193 Cerdanyola del Vallès, Spain; leirapaula.fernandez@irta.cat (L.F.-B.); jordi.rodon@irta.cat (J.R.); nuria.roca@irta.cat (N.R.); anna.pou@irta.cat (A.P.); guillermo.cantero@irta.cat (G.C.); cristina.lorca@irta.cat (C.L.-O.); julia.vergara@irta.cat (J.V.-A.); 2 IrsiCaixa AIDS Research Institute, 08916 Badalona, Spain; epradenas@irsicaixa.es (E.P.); SMarfil@irsicaixa.es (S.M.); btrinite@irsicaixa.es (B.T.); MParera@irsicaixa.es (M.P.); jcarrillo@irsicaixa.es (J.C.); nizquierdo@irsicaixa.es (N.I.-U.); bclotet@irsicaixa.es (B.C.); mnoguera@irsicaixa.es (M.N.-J.); jblanco@irsicaixa.es (J.B.); 3 IrsiCaixa AIDS Research Institute, Germans Trias i Pujol Research Institute (IGTP), Can Ruti Campus, 08916 Badalona, Spain; 4 Infectious Diseases and Immunity, Faculty of Medicine, University of Vic-Central University of Catalonia (UVic-UCC), 08500 Barcelona, Spain; 5 Centre de Recerca en Sanitat Animal (CReSA), Institut de Recerca en Tecnologies Agroalimentaries (IRTA), Campus de la UAB, 08193 Barcelona, Spain; 6 Departament de Sanitat i Anatomia Animals, Facultat de Veterinaria, Universitat Autònoma de Barcelona, 08193 Cerdanyola del Vallès, Spain

**Keywords:** SARS-CoV-2, B.1.617.2, Delta variant, variants of concern, COVID-19, dog, pets, transmission, reverse zoonosis

## Abstract

Several cases of naturally infected dogs with severe acute respiratory syndrome coronavirus 2 (SARS-CoV-2) have been reported despite the apparently low susceptibility of this species. Here, we document the first reported case of infection caused by the Delta (B.1.617.2) variant of concern (VOC) in a dog in Spain that lived with several household members suffering from Coronavirus Infectious Disease 2019 (COVID-19). The animal displayed mild digestive and respiratory clinical signs and had a low viral load in the oropharyngeal swab collected at the first sampling. Whole-genome sequencing indicated infection with the Delta variant, coinciding with the predominant variant during the fifth pandemic wave in Spain. The dog seroconverted, as detected 21 days after the first sampling, and developed neutralizing antibodies that cross-neutralized different SARS-CoV-2 variants. This study further emphasizes the importance of studying the susceptibility of animal species to different VOCs and their potential role as reservoirs in the context of COVID-19.

## 1. Introduction

SARS-CoV-2 is responsible for the ongoing Coronavirus Infectious Disease 2019 (COVID-19). Since the initial outbreak in Wuhan (China) at the end of 2019, the World Health Organization (WHO) has reported more than 270 million cases of COVID-19, causing approximately 5.3 million deaths worldwide (WHO, accessed on 15 December 2021, https://covid19.who.int/) [1]. The massive and rapid transmission of SARS-CoV-2 has led to the emergence of several viral variants, some of which have raised high concern due to their impact on transmissibility, mortality and their putative capacity to escape from immune responses generated after infection or vaccination [2].

To date, there have been four globally recognized variants of concern (VOC), including Alpha or lineage B.1.1.7 (first described in the UK) [3], Beta or lineage B.1.351 (initially identified in South Africa) [4], Gamma or lineage P.1 (first described in Brazil) [5], and Delta or lineage B.1.617.2 (initially detected in India) [6]. The appearance of these VOCs resulted from the accumulation of mutations along the whole SARS-CoV-2 genome; however, those located in the gene that codes for the spike (S) protein have been emphasized because the S protein mediates viral entry into target cells [7,8,9]. The binding of the S protein to the angiotensin-converting enzyme 2 (ACE2), identified as the main cellular receptor for SARS-CoV-2 entry, determines infectivity, tropism and host range [10].

Bats are believed to be the original host of SARS-CoV-2; however, it is still unclear whether an intermediate host eventually transferred the virus to humans [10,11]. Since the beginning of the pandemic, several domestic and wild animals have shown to be susceptible to SARS-CoV-2 infection [12,13]. Moreover, reverse zoonosis episodes have been documented on farms, in zoos and in familiar households [14,15,16,17]. Recently, by infecting wild-type mice, it has been shown that some SARS-CoV-2 variants display an increased virulence in humanized ACE2 transgenic mice and a broadened host range [18,19]. However, little is known about the capabilities of VOC in terms of causing differential virulence or expanded infection tropism in animal species undergoing natural SARS-CoV-2 infection.

Since pet species are at high risk of SARS-CoV-2 exposure due to close contact with their owners, it is crucial to monitor VOC transmission events and understand whether they could pose a higher risk for these animal populations. SARS-CoV-2 transmission from humans to dogs has been described in several parts of the world with early pandemic variants [16,20,21], and recently with both the Alpha (B.1.1.7) [22,23] and the Delta (B.1.617.2) VOCs [24]. Here, we document the first reported case of a symptomatic dog infected with the Delta VOC (B.1.617.2) in Spain, which occurred during the fifth wave of SARS-CoV-2 infection. This dog was living with owners who had been diagnosed with COVID-19 one week prior to the dog developing clinical signs, confirming that transmission of the Delta variant from human to dogs is possible and that animals may develop mild clinical signs similar to those in humans.

## 2. Materials and Methods

### 2.1. Clinical Evaluation and Sample Collection

**Figure 1:**
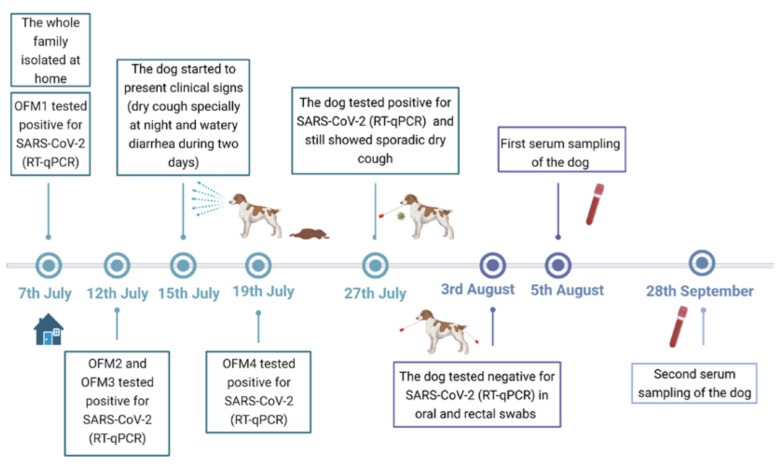
Chronological events relating to the SARS-CoV-2 Delta variant infection of the dog. The timeline shows when the owner’s family members and the dog tested positive, as well as the dates that samples were collected. Abbreviations: Owner Family Members (OFM). Reverse transcription quantitative-polymerase chain reaction (RT-qPCR).

By mid-July 2021, a 13-year-old female Breton dog developed respiratory and digestive signs coinciding with the timing of their owners suffering from COVID-19 (Figure 1). Since mild respiratory signs were still present approximately two weeks later, an oropharyngeal swab was collected on 27 July 2021. On 3 August 2021, an oropharyngeal and a rectal swab were collected from the dog again despite it no longer displaying any clinical signs. Blood extraction for serological analysis was performed at two different time points: on 5 August 2021 (serum 1; Se1) and on 28 September 2021 (serum 2; Se2) (Figure 1). Blood samples were centrifuged at 1800× *g* for 10 min at 4 °C. The obtained sera were inactivated for 1 h at 56 °C and then stored at −20 °C until further use. All dog samples were collected at the Hospital Clínic Veterinari of the Universitat Autònoma de Barcelona (UAB, Bellaterra, Barcelona, Spain).

### 2.2. RNA Extraction and Detection by RT-qPCR

Oropharyngeal and rectal swabs were transferred into cryotubes containing 500 μL DMEM (Lonza, Basel, Switzerland) supplemented with 100 U/mL penicillin, 100 μg/mL streptomycin, and 2 mM glutamine (all from Gibco Life Technologies, Madrid, Spain) and finally vortexed. Viral RNA was extracted using the Indimag Pathogen kit (Indical Biosciences, Leipzig, Germany) on a Biosprint 96 workstation (Qiagen, Hilden, Germany), according to the manufacturer’s instructions. Detection of SARS-CoV-2 RNA was achieved following a previously described protocol targeting the envelope protein (E)-encoding gene [25] by an RT-qPCR method, applying minor modifications [26]. RT-qPCR was carried out using AgPath-ID^TM^ One-Step RT-PCR Reagents (Applied Biosystems, Life Technologies, Waltham, MA, USA). Amplification was achieved by using a 7500 Fast Real-Time PCR System (Applied Biosystems, Life Technologies, Waltham, MA, USA) (10 min at 50 °C; 10 s at 95 °C; 45 cycles of 15 s at 94 °C; and 30 s at 58 °C). Samples with a Cq value ≤ 40 were considered positive for SARS-CoV-2. To confirm the result, positive samples were also tested by RT-qPCR targeting the RNA-dependent RNA polymerase gene (RdRp) specific to the SARS-CoV-2 [25].

### 2.3. SARS-CoV-2 Genome Sequencing

For the positive samples, viral RNA was extracted and sequenced as previously described [27]. RNA was converted to cDNA with the PrimeScript^TM^ RT reagent kit (Takara Bio Europe SAS, Saint-Germain-en Laye, France) using a combination of oligo-dT and random hexamer methods, following the manufacturer’s protocol. cDNA was used for viral DNA enrichment using the ARTIC-CoV v3 PCR protocol and the Q5 Hot-start HF polymerase. The amplified PCR products were used for sequencing-ready library preparation with the Illumina DNA LibPrep kit (Illumina, San Diego, CA, USA). Next, sequencing-ready libraries were loaded onto the Illumina MiSeq platform and a 150 bp paired-end sequencing kit (300 cycles). Raw data analysis was performed using the viralrecon pipeline (https://nf-co.re/viralrecon/1.0.0 (accessed on: 15 December 2021)). Sequence reads were quality-filtered, and adapter primer sequences were trimmed using Trimmomatic [28]. Sequencing reads were then aligned against the reference Wuhan/Hu-1/20219 variant (NCBI accession number: NC_045512.2) using the Bowtie2 tool [29], while consensus genomic sequence was called from the resulting alignments using iVarsoftware at the 25% threshold. Genomic sequence was classified by the Pangolin lineage classification system (v.3.1.16, lineages version 18 October 2021). 

### 2.4. Neutralizing Antibody Detection by SARS-CoV-2 Receptor-Binding Inhibition ELISA

Seroneutralizing antibodies targeting RBD were measured with the GenScript cPass™ SARS-CoV-2 Neutralization Antibody Detection Kit (Genscript, the Netherlands), following the manufacturer’s protocol. Serum samples (1:10 diluted) were mixed with equal volumes of recombinant HRP-conjugated RBD and incubated for 30 min at 37 °C. Next, 100 μL of each diluted sample was transferred to 96-well plates pre-coated with a recombinant hACE2 receptor and incubated for 15 min at 37 °C. After four washing steps, the substrate solution (tetramethylbenzidine substrate, TMB) was incubated for 15 min at room temperature, after which the stop solution was added. Absorbance values were read at 450 nm in an automatic microELISA reader, and the percentage of inhibition of each sample was determined using the following formula: %inhibition = (1 − (OD450 sample/OD450 of negative control)) × 100. Each of the samples and controls was included in duplicate (SD ≤ 10%). Inhibition >30% was considered as a positive neutralization. 

### 2.5. SARS-CoV-2 Pseudoneutralization Assay

A pseudovirus-based neutralization assay of the Se2 sample was performed following a protocol previously described [30]. HIV reporter pseudoviruses expressing the SARS-CoV-2 S protein (from different VOCs) and Luciferase were generated. Control pseudoviruses were obtained by replacing the S protein expression plasmid with a VSV-G protein expression plasmid as reported previously [31]. For neutralization assay, 200 TCID50 of pseudovirus supernatant was preincubated with serial dilutions of the heat-inactivated plasma samples and then added onto ACE2 overexpressing HEK293T cells. After 48 h, cells were lysed with Britelite Plus Luciferase reagent (Perkin Elmer, Waltham, MA, USA). Luminescence was measured for 0.2 s with an EnSight Multimode Plate Reader (Perkin Elmer, Waltham, MA, USA). 

The neutralization capacity of the plasma samples was calculated by comparing the experimental RLU calculated from infected cells treated with each plasma to the max RLUs (maximal infectivity calculated from infected untreated cells), background minimal signal (non-infected cells), and expressed as percent neutralization: %Neutralization = (RLUmax − RLUexperimental)/(RLUmax − RLUmin) × 100. The SNT50 was calculated by plotting and fitting neutralization values and the log of plasma dilution to a 4-parameters equation in Prism 9.0.2 (GraphPad Software, San Diego, CA, USA). 

### 2.6. SARS-CoV-2 Neutralization Assay

A replicating-virus neutralization assay of the Se2 sample was performed as previously described [15]. The inactivated serum sample was first diluted at 1:10 and then 2-fold serially diluted in DMEM. Next, the diluted sample was mixed 1:1 with an isolate of SARS-CoV-2 (B.1 lineage) [27] and further incubated for 1 h at 37 °C. Each dilution mixture (in four replicates) was transferred onto Vero E6 (ATCC^®^ repository, Manassas, VA, USA, CRL-1586^TM^) cell monolayers containing 100 TCID50 of SARS-CoV-2 per well were cultured for 3 days at 37 °C and 5% CO_2_. Then, the cytopathic effect of the SARS-CoV-2 was measured using the CellTiter-Glo luminescent cell viability assay (Promega, Madison, WI, USA), following the manufacturer’s protocol. Luminescence was measured as relative luminescence units (RLU) in a Fluroskan Ascent FL luminometer (ThermoFisher Scientific, Waltham, MA, USA). The 50% serum virus neutralization titer (SNT50) was defined as the reciprocal dilution of the sample at which 50% of cells were protected. 

The dose–response curve of the serum sample was adjusted to a non-linear fit regression model calculated with a normalized logistic curve with variable slope. Uninfected cells and untreated virus-infected cells were used as negative and positive controls of infection for data normalization (%Neutralization = (RLUmax − RLUexperimental)/(RLUmax − RLUmin) × 100), respectively. All statistical analyses were performed with GraphPad Prism 8.4.3 (GraphPad Software, Inc, San Diego, CA, USA). 

## 3. Results

### 3.1. Clinical Follow-Up

On 15 July 2021, a 13-year-old female Breton dog developed respiratory signs, especially a dry cough at night, and digestive disorders (watery diarrhea for two days), at the time their owners suffered from COVID-19 (Figure 1). Some days before, on 5 July 2021, one of the owner’s family members (OFM1) was confirmed as a contact of a COVID-19-affected patient. Then, on 7 July 2021, OFM1 tested positive for SARS-CoV-2 by RT-qPCR and started developing symptoms including fever, dyspnea, and dizziness. He was finally hospitalized and diagnosed with bilateral pneumonia and severe respiratory insufficiency. The whole family was quarantined and also developed COVID-19-like symptomatology (fever, coughing and sneezing). Two of them (OFM2 and OFM3) were finally diagnosed with COVID-19 on 12 July 2021, while the last member (OFM4) of the family tested negative by RT-qPCR. It is noteworthy that OFM2 was vaccinated against SARS-CoV-2 three months before. On 19 July 2021, OFM4 was finally diagnosed with COVID-19 as well.

### 3.2. RNA Detection and SARS-CoV-2 Sequencing

The oropharyngeal swab from the dog collected on 27 July 2021 tested positive for SARS-CoV-2 UpE (Cq of 34.4) and RdRp (Cq of 35.8) genes by RT-qPCR. Eight days later, on 3 August 2021, the animal tested negative for the detection of SARS-CoV-2 RNA in both oropharyngeal and rectal swabs.

SARS-CoV-2 genomic RNA from the first oropharyngeal swab was successfully obtained (GISAID EPI ISL 6344510). The genomic sequence was classified as AY. 43, a sub-lineage within Delta/B.1.617.2 lineage.

### 3.3. Immune Response Elicited after SARS-CoV-2 Infection

**Figure 2:**
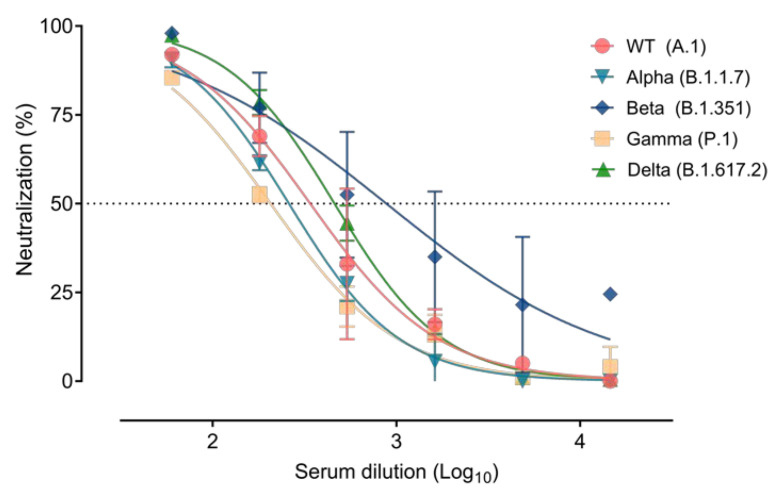
Neutralizing humoral responses developed after SARS-CoV-2 Delta variant infection of the dog. Neutralization assay of the serum sample 2 (Se2) against pseudoviruses expressing the spike proteins of the WT, the Alpha, the Beta, the Gamma and the Delta variants of SARS-CoV-2.

The dog elicited neutralizing antibodies against the RBD of SARS-CoV-2, as determined from serum samples collected 21 days after the display of clinical signs by the receptor binding inhibition assay. The Se1 sample showed an inhibition titer of 68.9% (SD ± 2.40%), and the Se2 sample (two and a half months after displaying the initial clinical signs) had an inhibition of 67.6% (SD ± 0.07%). We then evaluated the neutralization activity of Se2 using a pseudovirus assay; Se2 was able to neutralize the Alpha (SNT50 = 1/260), the Beta (SNT50 = 1/881), the Gamma (SNT50 = 1/207), the WT (SNT50 = 1/340), and the Delta (SNT50 = 1/460) variants (Figure 2). Moreover, titers of neutralizing antibodies against a replicating SARS-CoV-2 isolate (B.1 Pango lineage) were also confirmed (SNT50 = 1/135.8).

## 4. Discussion

This is the first case report of SARS-CoV-2 B.1.617.2 (Delta) VOC infecting a dog in Spain; the dog displayed respiratory and digestive clinical signs at the time of infection. It is speculated that the animal became infected by close contact with its owners since they were diagnosed with COVID-19 a week before the dog displayed clinical signs. 

Due to the large number of companion animals infected with SARS-CoV-2 since the beginning of the COVID-19 pandemic, SARS-CoV-2 infection in the dog in this study was suspected [14,16,32,33]. Low viral RNA loads in the oropharyngeal swab confirmed that the animal was infected with SARS-CoV-2; however, subsequent oropharyngeal and rectal swabs collected twenty days after the display of clinical signs were tested and were negative already. Whole-genome sequencing determined infection by the Delta (B.1.617.2) VOC, AY. 43 sub-lineage. Recently, a natural infection by the same variant was also reported in a dog in Kansas (USA) [24]. In addition, natural infections with the B.1.617.2 (Delta) variant have been reported in Asiatic lions, where a human-to-animal transmission was also suspected [34]. In this present study, samples from the owners of the studied dog were not available, but the existing epidemiological information suggests transmission from humans to the dog because the animal did not have other contacts. Furthermore, in agreement with our study, the majority of SARS-CoV-2 natural infections in companion animals, such as dogs and cats, have been reported in animals living in households with at least one SARS-CoV-2-infected owner [14,16,32,35]. In the present case, the infection occurred during the fifth wave of COVID-19 in Spain (July 2021), which was dominated by the Delta VOC variant [36].

The appearance of respiratory disorders in dogs was previously reported upon natural infection with the Alpha variant and was suspected with the Delta variant [23,24]. In fact, Doerksen et al. [24] could not unequivocally attribute observed clinical signs of the dog to the Delta VOC since the animal had other underlying conditions. Importantly, the dog in the present study also displayed digestive clinical signs for two days, which are compatible with SARS-CoV-2 infection. However, it is not possible to rule out the presence of concomitant infections or other conditions affecting the dog at the time of the clinical signs. The low viral load found in the animal in the first oropharyngeal swab sampling suggests that it was already clearing the virus because the clinical signs had started almost two weeks before. In any case, the clinical signs disappeared after the SARS-CoV-2 infection was cleared, supporting the effect of this virus in the clinical condition of the dog. Furthermore, seroconversion to SARS-CoV-2 was confirmed 21 days after the appearance of the clinical signs, and similar levels of antibodies were maintained after two and a half months. Despite the fact that the Delta variant was the variant infecting the dog, the humoral response generated was able to cross-neutralize against the other viral variants in vitro (Alpha, Beta, Gamma, and the first variant reported in Wuhan). Neutralizing responses were developed at similar levels against all the tested SARS-CoV-2 variants. However, the titers of neutralizing antibodies were not high when compared to severely infected human patients [30] and were similar to those that have been described in dogs infected with SARS-CoV-2 [16,22,23]. 

In summary, the present study confirms that the SARS-CoV-2 Delta VOC (B.1.617.2) can spread to animals exposed to COVID-19 environments and can potentially cause clinical infection. Here we reported the infection of a dog living in contact with COVID-19-positive family members. The dog displayed respiratory and digestive clinical signs during the time of infection, subsequently cleared the virus within twenty days and developed neutralizing responses to different SARS-CoV-2 variants. This case highlights the importance of studying the potential difference of host susceptibility upon transmission of SARS-CoV-2 VOC from humans to animals.

## Data Availability

All data are available upon request to the authors.
